# Complete Genome Sequence of Leptospira kobayashii Strain E30, Isolated from Soil in Japan

**DOI:** 10.1128/MRA.00907-21

**Published:** 2021-11-11

**Authors:** Ryo Nakao, Toshiyuki Masuzawa, Shuichi Nakamura, Nobuo Koizumi

**Affiliations:** a Laboratory of Parasitology, Department of Disease Control, Graduate School of Infectious Diseases, Faculty of Veterinary Medicine, Hokkaido University, Hokkaido, Japan; b Laboratory of Microbiology and Immunology, Faculty of Pharmaceutical Sciences, Chiba Institute of Science, Chiba, Japan; c Department of Applied Physics, Graduate School of Engineering, Tohoku University, Miyagi, Japan; d Department of Bacteriology I, National Institute of Infectious Diseases, Tokyo, Japan; University of Maryland School of Medicine

## Abstract

The spirochete bacterium Leptospira kobayashii is a recently designated species of the genus *Leptospira*. Here, we report the complete genome sequence of L. kobayashii strain E30, consisting of two circular chromosomes and two plasmids.

## ANNOUNCEMENT

Leptospira kobayashii strain E30 was isolated from soil in Japan (1, 2). The bacterium has a long, thin spiral cell body with hook-shaped ends, which is a typical morphology for the members of the genus *Leptospira*, family *Leptospiraceae*, and order *Spirochaetales* (3). The strain is a saprophyte; it grows at 13°C and with 8-azaguanine (final concentration, 225 μg/ml) at 30°C and does not infect mice (4, 5). The bacterium is motile, but its smooth swimming is induced by visible light exposure (6).

L. kobayashii strain E30 was cloned from the primary culture that had been cryopreserved at −80°C at Chiba Institute of Science (Chiba, Japan) by colony formation using a plate of Ellinghausen-McCullough-Johnson-Harris (EMJH) medium (1% agar) and then was maintained using liquid EMJH medium at 30°C (3). Genomic DNA of L. kobayashii strain E30 was extracted using the Wizard genomic DNA purification kit (Promega, Madison, WI) and Genomic-tips (Qiagen, Germany) for Illumina and Nanopore sequencing, respectively. The Illumina sequencing library was prepared with 200 ng of total DNA using a TruSeq Nano DNA sample preparation kit (Illumina, San Diego, CA) following the manufacturer’s instructions. A total of 5,615,056 paired-end sequencing reads, with an average length of 298.6 bp, were obtained on the MiSeq platform using the MiSeq reagent kit v3 (Illumina). The Nanopore sequencing library was prepared with 1,000 ng of total DNA, without shearing, using a native barcoding expansion kit (EXP-NBD104), and fragments of >3 kb were selected using a ligation sequence kit (SQK LSK 109) (Oxford Nanopore Technologies, Oxford, UK) following the manufacturer’s instructions. The libraries were loaded onto R9.4.1 flow cells and run on a GridION system (Oxford Nanopore Technologies). The resulting fast5 reads were base called using Guppy v4.4.0 (Oxford Nanopore Technologies), and a total of 39,278 reads, with an average length of 10,007.3 bp, were obtained.

Raw Illumina reads were trimmed using Sickle v1.33 (https://github.com/najoshi/sickle) with a minimum quality value (QV) score of 20 and a minimum nucleotide length of 127 nucleotides, based on QV scores. The Nanopore reads were filtered using Filtlong v0.2.0 (https://github.com/rrwick/Filtlong) with a minimum nucleotide length of 1,000 nucleotides to yield 400,000,000 bp, and errors were corrected using Canu v1.8 (7) with default settings. The filtered Illumina and Nanopore reads were *de novo* assembled using Unicycler v0.4.7 (8) with default parameters. The assembled contigs were visualized using Bandage v0.8.1 (9) to detect circular genomes, and the completeness of the genome was assessed with CheckM v1.0.12 (10). The resulting complete genome was annotated by the DDBJ Fast Annotation and Submission Tool (DFAST) v1.2.13 with default parameters (11).

The genome of L. kobayashii strain E30 consists of two circular chromosomes (3,985,339 and 274,191 bp in length) and two plasmids (45,413 and 5,386 bp in length). Chromosome I contains 3,609 coding sequences, 6 rRNAs (two each of 5S, 16S, and 23S rRNAs), and 36 tRNAs, while chromosome II contains 250 protein-coding genes (Table 1). Plasmids pE30-1 and pE30-2 contain 43 and 6 protein-coding genes, respectively. The data presented here will facilitate comparative genomic analyses of the genus *Leptospira* and expand our understanding of the genetic diversity of *Leptospira* species and the molecular mechanisms of the photoresponsive motility of L. kobayashii.

**TABLE 1:** Summary of the genome of Leptospira kobayashii strain E30

Genetic element	Size (bp)	Coverage (×)	GC content (%)	No. of coding sequences	No. of rRNAs	No. of tRNAs	Accession no.
Chromosome 1	3,985,339	74	40.7	3,609	6	36	AP025028
Chromosome 2	274,191	68	40.9	250	0	0	AP025029
pE30-1	45,413	27	39.3	43	0	0	AP025030
pE30-2	5,386	229	44.7	6	0	0	AP025031
Total	4,310,329		40.7	3,908	6	36	

### Data availability.

The complete genome sequence was deposited in DDBJ/EMBL/GenBank under accession numbers AP025028, AP025029, AP025030, and AP025031. The raw sequence data were deposited in the SRA under accession numbers DRR124442 (Illumina reads) and DRR315250 (Nanopore reads).
