# Correction for Nakao et al., “Complete Genome Sequence of Leptospira kobayashii Strain E30, Isolated from Soil in Japan”

**DOI:** 10.1128/MRA.01169-21

**Published:** 2021-12-16

**Authors:** Ryo Nakao, Toshiyuki Masuzawa, Shuichi Nakamura, Nobuo Koizumi

**Affiliations:** a Laboratory of Parasitology, Department of Disease Control, Graduate School of Infectious Diseases, Faculty of Veterinary Medicine, Hokkaido University, Hokkaido, Japan; b Laboratory of Microbiology and Immunology, Faculty of Pharmaceutical Sciences, Chiba Institute of Science, Chiba, Japan; c Department of Applied Physics, Graduate School of Engineering, Tohoku University, Miyagi, Japan; d Department of Bacteriology I, National Institute of Infectious Diseases, Tokyo, Japan

## AUTHOR CORRECTION

Volume 10, no. 45, e00907-21, 2021, https://doi.org/10.1128/MRA.00907-21. After publication, the authors became aware that the sequences of pE30-2 were derived from the PhiX sequencing control. We apologize for the inconvenience caused.

Page 1, abstract: “two circular chromosomes and two plasmids” should read “two circular chromosomes and one plasmid.”

Page 2, Table 1: line 4 should be deleted. The total size (bp) should read “4,304,943,” and the total number of coding sequences should read “3,902.”

Page 2, line 2: “two plasmids (45,413 and 5,386 bp in length)” should read “one plasmid (45,413 bp in length).”

Page 2, lines 4 and 5: “Plasmids pE30-1 and pE30-2 contain 43 and 6 protein-coding genes, respectively.” should read “Plasmid pE30-1 contains 43 protein-coding genes.”

Page 2, line 10: “accession numbers AP025028, AP025029, AP025030, and AP025031” should read “accession numbers AP025028, AP025029, and AP025030.”

